# A comparative study of mono ethylene glycol economic production via different techniques

**DOI:** 10.1038/s41598-024-77713-y

**Published:** 2024-11-17

**Authors:** Walaa M. Shehata, Taha G. Nady, Fatma K. Gad, Abeer M. Shoaib, Ahmed A. Bhran

**Affiliations:** 1 Petroleum Refining and Petrochemical Engineering Department Faculty of Petroleum and Mining Engineering, Suez University, P.O. Box: 43221, Suez, Egypt; 2 United Gas Derivative Company, Port Said, Egypt; 3 Chemical Engineering Department College of Engineering, Imam Mohammad Ibn Saud Islamic University, Riyadh, Saudi Arabia

**Keywords:** MEG Technology, Ethylene Oxide, Ethylene Carbonate, Economic Analysis, Chemical engineering, Energy

## Abstract

Mono-ethylene glycol (MEG) is a high-volume chemical intermediate used as a raw material for a variety of chemical products. It could also be used as a hydrate inhibitor in natural gas. Recently, the importance of MEG has been increased due to its usage as a supporting emulsifier in diesel engines to reduce NOx and soot emissions, in addition to its usage as an additive to dual fuel diesel engines. The increase consumption of MEG in wide range of applications leads to the search for the most efficient, environmental friendly and cost effective technique to produce more quantities of it. MEG is most commonly manufactured via the hydration of ethylene oxide (EO). In this work, two different technologies of EO hydration to produce MEG are compared; the direct hydration of EO with water and the indirect hydration through the usage of ethylene carbonate (EC) as an intermediate. Comparative economic and environmental impact assessments were performed based on plant-scale simulations (per 600,000 tons per year of MEG produced) of the two hydration technologies using Aspen HYSYS version 11 simulation software. Economic analysis showed that the utilities’ energy consumption for direct hydration process is significantly higher than for indirect hydration by 279 megawatts. On the other hand, the environmental impact assessments showed that GHG emissions from natural gas power generation from utilities from direct hydration are three times greater than GHG emissions from indirect hydration. This leads to indirect hydration of ethylene oxide through ethylene carbonate formation being considered economically and environmentally preferable compared to the direct hydration process of ethylene oxide.

## Introduction

Strict environmental regulations and population growth lead the researchers to search for cleaner and energy-efficient manufacturing processes in chemical industries. Toward this goal, this paper targets the manufacturing of one of the world’s largest bulk chemical intermediates, mono-ethylene glycol (MEG) or ethylene glycol (EG); it is considered as the simplest diol among the three di-hydroxy alcohols; which are mono, di, and tri ethylene glycol. It was first developed in 1859 through the saponification of ethylene glycol diacetate (EGD) with potassium hydroxide, by a French chemist called Charles-Adolphe Wurtz^1^. Ethylene glycol (EG) is considered an incredibly important organic compound since it is widely used in lots of catalytic and non-catalytic chemical industries such as anti-freezing, liquid detergent, pharmaceuticals, explosives, hydraulic brake fluids, engine coolants, and hydrate inhibitors used for the recovery of subsea oil and gas^2,3^. Moreover, it is used as an intermediate compound in many petrochemical industries^4,5^; synthesis of resins, plastics, textiles^6,7^, biodegradable polyester fibers, cosmetic products^8,9^, plasticizers, fabric, solvents for paints polyethylene terephthalate (PET) resin used in fabricating plastic bottles for soft drinks and other down-stream products^10,11^. The need for MEG has been extended over the years to nonconventional uses; it is found recently that it could be used in diesel engines as an auxiliary emulsifier in order to maximize engineperformance and minimize NOx, CO and soot emissions^12,13^. Widyanto and Suputra Wiguna^14^ found that it has significant corrosion inhibition effect on low carbon steel pipelines in acidic environment with increased temperature. It is found that EG global demand in 2014 had reached 25 million tons and annually increases by about 5% due to the huge increase in standards of living in areas of high population like India, China,, and South America^15^.

Due to this wide and attractive commercial applications, a diversity of chemical systems and research interests have considerably focused on EG synthesis either from fossil fuels or biomass-based resources^4^. MEG was commonly synthesized via non-catalytic hydration of ethylene oxide (EO) by water at an elevated temperature^16^. EO is synthesized by the oxidation of ethylene, either in the vapor phase or liquid phase. However, many other byproducts are produced, like di- and tri-ethylene glycols, in addition to small amounts of tetra-ethylene and heavier glycols. Selective production of MEG in this process requires increasing water-to-ethylene ratio; this consequently results in higher energy consumption and capital investment for water evaporation or distillation^17^. Hence, many research works focused on exploring new and selective catalysts have aimed to reduce the required molar water to EO ratio and introduced excellent reviews for different needed catalysts^18–20^. Van et al. studied the effect of catalytic hydration of ethylene oxide compared to thermal hydration for MEG formation^21^, while Ranu and Banerjee studied the usage of ionic liquid catalysts as a reaction medium in Michael addition^22^.

Other alternative routes have been investigated over the years to increase the selectivity of MEG and reduce energy costs. One of these routes involves the formation of ethylene carbonate (EC) as an intermediate for MEG production, which is produced by the reaction of ethylene oxide with carbon dioxide. Hydrolysis of EC with a little amount of water results in highly selective production of MEG. This route promises the production of negligible amounts of higher glycols in addition to a considerable reduction in steam consumption^3,23^. This process is preferentially carried out in the presence of a catalyst; many research works have focused on the suitable catalyst for such process^24,25^. Additionally, some patents have been awarded for the production of ethylene glycol via different proposed processes^26–28^. However, no successful commercial applications have been reported so far^17^. An alternative route for EG synthesis, called one-pot synthesis, has been introduced recently and studied in many research works; it is based on the direct catalytic reaction of ethylene with hydrogen peroxide^3,29,30^. Liang et al.^31^ have studied the electrocatalytic conversion of ethylene to ethylene oxide mediated by halide oxidation. Moreover, Simultaneous process optimization and heat integration for ethylene-to-ethylene oxide process have been studied by Wang et al.^32^. On the other hands, the need for MEG as inhibitor in deep marine gas plants and the future requirements of it have been studied recently^33^. Hence, the preparation of EG to overcome the wide range of marketing and industrial requirements varies widely; the selection of the best route depends on many parameters, like selectivity, reactivity, cost, and, catalyst availability^34^.

Very recently, some research have been rooted toward the production of MEG from renewable resources^35,36^, in addition to the studies which focused on the economical assessment of such roots and their potential in industrial biotechnology^37,38^, however, the production of it from biomass still could not satisfy the huge amounts of requirements and the process is not cost effective. Hence, the conventional technologies for the production of EG is still needing to be further studied and upgraded in order to satisfy the huge and diverse usage of such chemicals.

The glycol production methods based on production of EG from ethylene oxide and water described above are of industrial importance. Although these methods are simple, they have some major drawbacks; they still have low selectivity for monoethylene glycol production since its selective synthesis requires a high H_2_O/EO ratio to prevent the formation of higher grades of glycol. For example, the MEG produced can react with EO to form diethylene glycol (DEG), and EO can react with DEG to form triethylene glycol (TEG). Moreover, energy consumption is also high in the distillation tower because there is a large amount of excess water used to hydrate the ethylene oxide. Accordingly, more research is required to improve MEG synthesis processes. On the other hand, the production of EG from ethylene oxide and carbon dioxide via the formation of ethylene carbonate as a feedstock (indirect hydration) can contribute to greater selectivity of MEG compared to di- and tri-ethylene glycols. In addition, the use of carbon dioxide as a raw material along with ethylene oxide is significant as environmental impacts. Its use contributes to reducing greenhouse gases in the atmosphere that contribute to global warming and climate change. But available data on this process are missing in the literature. The operating conditions and detailed structural design configuration have not been covered in previous studies. The final greenhouse gas emissions from this process have not been calculated. In addition, it is not known whether this process is economically and environmentally better or not compared to the direct hydration process.

In this work, an EG model is presented for the indirect hydration of ethylene oxide and carbon dioxide through the formation of ethylene carbonate as an intermediate. In addition, a comparison was made between this process and the direct hydration process for producing EG from ethylene oxide and water. The two EO hydration technologies (direct and indirect hydration) are a detailed design for producing ethylene glycol with increased selectivity for MEG. In addition, an economic and environmental comparison was made between the two glycol production technologies, and the best technology was chosen according to economic and environmental considerations regarding greenhouse gas emissions.

## Methodology

This research presents the initial design of a plant for the production of 600,000 tons/year of ethylene glycol from ethylene as a feedstock using direct and indirect hydration of ethylene oxide. The goal of this work is to obtain the best effective technology for producing ethylene glycol from ethylene, which is less expensive and has an environmentally friendly effect in terms of carbon emissions. To achieve this, modeling and comparison between the two hydration technologies were used and studied through economic and greenhouse gas emissions evaluation. Modeling and design were carried out in two parts. The first part involves the oxidation of ethylene to ethylene oxide. The second part involves the hydration of ethylene oxide to produce MEG by two alternative techniques; direct hydration with water and indirect hydration through the formation of ethylene carbonate as an intermediate. The results of the first and second parts were studied and compared to obtain the best, lowest cost and environmentally friendly model for producing ethylene glycol from ethylene.

This paper presents the preliminary design of a 600,000 tons/year production plant of ethylene glycol from ethylene as a feedstock using direct and indirect hydration of ethylene oxide. The aim of this work is to obtain the most effective technology for producing ethylene glycol from ethylene which is less expensive and has an environmentally friendly effect in terms of carbon emissions. To achieve this, modeling and comparison between the two hydration technologies were used and studied through economic and greenhouse gas emissions evaluation. Modeling and design were carried out in two parts. The first part involves the oxidation of ethylene to ethylene oxide. The second part involves the hydration of ethylene oxide to produce MEG by two alternative techniques; direct hydration with water and indirect hydration through the formation of ethylene carbonate as an intermediate. The results of the first and second parts were studied and compared to obtain the best, least expensive and environmentally friendly model for producing ethylene glycol from ethylene.

### Simulation assumptions

The simulation software HYSYS version 11 was used in the study. HYSYS is used as a versatile process simulation and problem solving tool. However, simulation and modelling using HYSYS software is used as a technical solution to provide significant economic benefits throughout the process-engineering life cycle. This software can provide flow information for mass and energy flows that are used in design specifications and cost estimates for various process equipment such as pumps, heat exchangers, and distillation columns. Engineering studies using HYSYS simulation can build a new design for chemical engineering processes and identify design changes that will improve plant performance, reliability and safety of plant operations. Furthermore, if these design changes are identified early, they can be implemented at low cost and provide significant savings during the life of the plant^39^. Table 1 describes the assumptions used in the HYSYS software to perform the modelling and simulation of the oxidation of ethylene to ethylene oxide and the two hydration processes of ethylene oxide to ethylene glycol.

**Table 1:** Specifications and assumptions used in simulation of ethylene-to-ethylene oxide and hydration of ethylene oxide to MEG processes^4,39–42^.

Process	Assumptions
General assumption for all simulation processes	Fluid package is glycol package
	Reactions are conversion stoichiometric reactions
	-All mixers are mapped as static mixers
	-All heat exchangers are mapped as TEMA heat exchanger
	-Al l reboilers are kettle type and condensers are TEMA type
Oxidation of ethylene to ethylene oxide process in the liquid phase (E/EO)	Hydrogen peroxide reactor is conversion reactor and mapped as Fixed tube sheet heat exchangers with selectivity of 82% and 18% for H_2_O_2_ and H_2_O, respectively.
	Ethylene oxidation reactor is conversion reactor and mapped as Fixed tube sheet heat exchangers (three reactors in parallel) with 99% EO product selectivity.
	-Hydrogen peroxide distillation tower is 12 sieve trays with condenser pressure of 1572 kPa and reboiler pressure of 3043 kPa. - Ethylene oxide distillation tower is 14 sieve trays with condenser pressure of 1965 kPa and 2063 kPa for reboiler pressure.
Direct hydration of ethylene oxide to monoethylene glycol process (EO/MEG)	Ethylene oxide reactor is conversion reactor and mapped as Fixed tube sheet heat exchangers with selectivity of 85% and 15% for MEG and di and tri, ethylene glycol, respectively.
	-MEG hydration tower is distillation tower with 15 sieve trays with condenser pressure of 15 kPa and reboiler pressure of 20 kPa. -MEG purification tower is distillation tower with 26 sieve trays with condenser pressure of 12.5 kPa and reboiler pressure of 13 kPa.
Indirect hydration of ethylene oxide to monoethylene glycol process through formation of ethylene carbonate as an intermediate (EO/MEG)	-Ethylene carbonate reactor is conversion reactor and mapped as Fixed tube sheet heat exchangers with selectivity of 98% (2 reactors in parallel)
	-MEG reactor is conversion reactor and mapped as Fixed tube sheet heat exchangers with selectivity of 90% and 10% for MEG and di and tri, ethylene glycol, respectively [40].
	-MEG hydration tower is distillation tower with 12 sieve trays with condenser pressure of 201.3 kPa and reboiler pressure of 301.3 kPa. -MEG purification tower is distillation tower with 14 sieve trays with condenser pressure of 108.2 kPa and reboiler pressure of 251.3 kPa.

HYSYS based process flowsheets for oxidation of ethylene to ethylene oxide and direct and indirect hydration of ethylene oxide to MEG were developed using published literature and patents^4,40–42^.

### **Economic assessment analysis**

The cost analysis is limited to the equipment presented in the flowsheets which are used to simulate the oxidation of ethylene in the liquid phase and the hydration of ethylene oxide (direct and indirect) processes. The cost estimate includes the installation cost of the considered equipment. However, it does not include other costs such as land procurement, preparation, service buildings, or owners’ costs.

In this study, the economic evaluation is conducted using the Aspen HYSYS process economic analyzer (formerly named Icarus Process Evaluator). Aspen’s economic assessment is a comprehensive economic tool within the Aspen HYSYS software that allows process engineers to quickly estimate the operating and capital costs for the processes being studied. The fixed capital investment encompasses the expenses for acquired equipment, offsite installed capacity, and direct installation expenditures. Production costs include raw material costs and utility expenses costs. Costs of raw materials, products, catalysts and solvents are derived from various sources including Chemicals Market Report. Operating costs associated with cooling water, electricity, and steam for both processes were estimated based on energy balance calculations achieved by Aspen HYSYS.

The return on investment (ROI) and payback period refer to the time required to recover the capital cost of a plant and are calculated according to Eqs. 1 and 2^43–45^.

1 $$\:\text{R}\text{O}\text{I}=\:\frac{\text{A}\text{v}\text{e}\text{r}\text{a}\text{g}\text{e}\:\text{y}\text{e}\text{a}\text{r}\text{l}\text{y}\:\text{n}\text{e}\text{t}\:\text{p}\text{r}\text{o}\text{f}\text{i}\text{t}}{\text{T}\text{o}\text{t}\text{a}\text{l}\:\text{c}\text{a}\text{p}\text{i}\text{t}\text{a}\text{l}\:\text{c}\text{o}\text{s}\text{t}}$$

2 $$\:\text{P}\text{a}\text{y}\text{b}\text{a}\text{c}\text{k}\:\text{p}\text{e}\text{r}\text{i}\text{o}\text{d}=\:\frac{1}{\text{R}\text{O}\text{I}}$$

### Environmental impact analysis

The simulation software Hysys version 11 is used to model the studied processes (oxidation of ethylene to ethylene oxide, direct hydration of ethylene oxide to monoethylene glycol, and indirect hydration of ethylene oxide to monoethylene glycol through the formation of ethylene carbonate as an intermediate). Furthermore, HYSYS version 11 contains US specific datasets related to environmental impacts arising from crude oil extraction, coal and natural gas-based power generation, etc., and includes computer datasets developed by the U.S. Environmental Protection Agency (USEPA)^46^ to conduct the United States’ environmental assessment^46,47^. Quantitative information on the various mass and energy currents associated with the studied processes was obtained from the HYSYS software. The cumulative environmental impacts from potential emissions from these streams were compared in relation to various potential environmental impacts of global warming. HYSYS contains US-specific datasets for emissions of greenhouse gases (GHGs) arising from natural gas-based power generation and the consumption and generation of natural gas-based utilities (cooling water, steam, and refrigerants). Quantitative information on greenhouse gases arising from cold and hot utilities and natural gas-based power generation associated with the studied processes was obtained from Aspen HYSYS simulations. These gases contribute to global warming and climate change. Cumulative greenhouse gas emissions from the studied processes were compared annually with respect to the environmental impact on global warming potential. In addition to the HYSYS calculated GHG emissions for utilities and power generation, the GHG emissions from production of the raw materials used in the study were added to the cumulative GHG emissions for each process studied. For example, the raw materials for the process of ethylene oxidation to ethylene oxide are ethylene, hydrogen, and oxygen. The GHG emissions during ethylene-based ethane cracking are added to the total GHG emissions from the oxidation of ethylene to the ethylene oxide process. The datasets in HYSYS are based on the use of natural gas as a feedstock for utilities and power generation, so it is assumed that ethylene and hydrogen production processes rely on natural gas as a feedstock for the two feedstocks. Emissions of GHSs from cracking ethane (derived from natural gas) to produce ethylene and hydrogen as a byproduct are added to the cumulative greenhouse gas emissions from the process of oxidation of ethylene to ethylene oxide. Results obtained from the HYSYS program for greenhouse gas emissions are expressed as CO_2_ equivalent mass per year.

The present work presents a quantitative environmental assessment of direct and indirect hydration of ethylene oxide processes using HYSYS software. Greenhouse gas emissions resulting from the two comparative processes were studied. Hotspots of greenhouse gas emissions from the two operations were identified.

## Ethylene Glycol Process Description

**Fig. 1:**
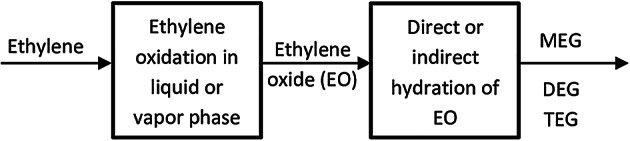
Block diagram of ethylene glycol production.

A simplified block diagram of the EG production process from ethylene is shown in Fig. 1. It is divided into two main sections:

- Ethylene oxidation in the liquid phase or vapor phase for producing ethylene oxide.
- Direct or indirect hydration of EO for producing EG.

A current petrochemical plant produces approximately 300,000-400,000 tons of ethylene per year. This plant is planning to use this ethylene product to produce 600,000 tons/year of MEG as a new project. Thus, the aim of the present study is directed to investigate the MEG production techniques and select the best technique to provide the annual amount of MEG required using ethylene as the feedstock. MEG production is based on two successive processes, the oxidation process of ethylene to get EO and the direct or indirect hydration process of EO to get MEG.

### Ethylene oxidation

As presented in Ghanta^40^, there are two types of ethylene oxidation for producing ethylene oxide required for producing MEG; oxidation in the vapor phase and oxidation in the liquid phase. The oxidation in the vapor phase depends on the direct oxidation of ethylene gas with oxygen while the oxidation in the liquid phase, the oxidant is hydrogen peroxide (H_2_O_2_). They show that the liquid-phase oxidation process of ethylene to produce EO is economically preferred over the vapor-phase process for many reasons. The vapor phase reactions need a higher temperature within the range of 160–200 ^o^C which requires a shell equipped with a heating steam system to maintain the high working temperature, unlike the liquid phase reactions which performed at a lower temperature within the range of 35–40 °C. It is clear that for similar EO production capacity, the quantity of ethylene consumed is higher in the case of the vapor phase process by 15%. This consequently indicates that the 15% excess of ethylene used in the vapor phase oxidation process is burned to CO_2_ and water^48–50^. The higher amount of ethylene used in the vapor phase oxidation process leads to a feedstock loss. The separation section in the vapor phase oxidation process involves an absorber to absorb the ethylene oxide and a stripper for removing water, unlike the liquid oxidation reaction which requires only a distillation column to separate water and hydrogen peroxide. Moreover, the CO_2_ recovery section in the vapor phase oxidation is an added value in the capital cost and this section is not needed in the liquid phase oxidation. The material of construction for the reactors in the vapor phase oxidation process is carbon steel. However, in the case of the liquid phase ethylene oxidation process the reactor is constructed of stainless steel (SS-304) to minimize metal-catalyzed decomposition of H_2_O_2_.

Regarding safety consideration of the vapor phase oxidation process, there are safety concerns regarding the flammability limits of the EO, O_2_, and ethylene. This could be handled by minimizing the conversion per pass by installing three reactors instead of only one reactor and adding inert gases to dilute the concentration of flammable gases. However, the addition of inert gases will lead to consume more energy in recompression and increase the size of the equipment and recycling lines and this, in turn, will increase the equipment and utility costs. On the other hand, for the liquid phase oxidation process, there are not safety issues due to the presence of the stable oxidant H_2_O_2_ and the vapors are free of oxygen.

This study considers the modelling, simulation and economic evaluation of liquid phase oxidation process used to produce EO as well as the two considered hydration (direct and indirect) techniques used to convert EO to MEG.

#### Ethylene oxidation in the liquid phase

**Fig. 2:**
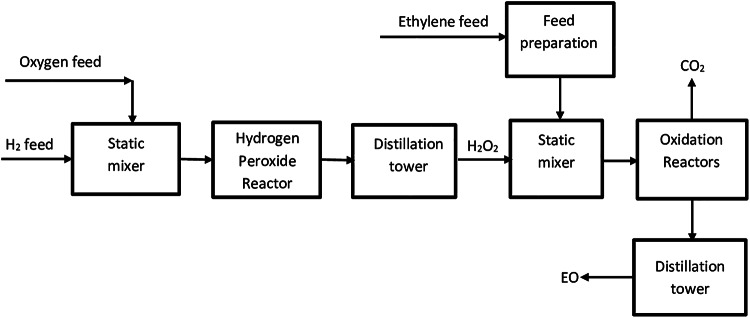
Block diagram of Ethylene oxidation for producing EO in the liquid phase using H_2_O_2_ as oxidant.

Ethylene oxide is prepared by the selective liquid-phase oxidation of ethylene by hydrogen peroxide (H_2_O_2_) in the presence of a homogeneous catalyst, which is methyl-trioxorhenium (MTO). The liquid-phase reaction mixture, including water is mixed with the compressed ethylene gas (4000 to 5000 kPa) in the presence of hydrogen peroxide as an oxidant, methanol and MTO. Pyridine N-oxide (PyNO) is used as a promoter in the temperature range of 20–40 °C. It is worth to mention that methanol could be replaced by t-butyl alcohol to serve as a solvent. The MTO catalyst helps in transferring the oxygen atom from H_2_O_2_ to ethylene, then, ethylene oxide is produced selectively. Carbon footprint reduction and feedstock conservation could be obtained by burning elimination. It is noticed that hydrogen peroxide remains stable at these conditions, in which the vapor phase is free of oxygen. Additionally, the operating pressure helps in keeping the produced ethylene oxide dissolved in the liquid phase; so, problems of EO flammability when it is in vapor phase could be avoided. The process is considered safe as it avoided the presence of oxygen and ethylene oxide in the vapor phase^40,48^. Figure 2 is a block diagram of ethylene oxide production in the liquid phase.

In this work, the oxidant H_2_O_2_ was prepared as a previous step for the ethylene oxidation process. Where oxygen gas is mixed with hydrogen, then the oxidation reaction takes place in the hydrogen peroxide reactor to produce H_2_O_2_ according to Eq. 3^40^.

3 $$\:{\text{H}}_{2}+{\text{O}}_{2}\:\:\to\:\:\:\:{\text{H}}_{2}{\text{O}}_{2}$$

In the H_2_O_2_ process, synthesis of H_2_O_2_ is facilitated by palladiumdinitrate (Pd(NO_3_)_2_) catalyst supported on a steel monolith in a fixed bed reactor. Under conditions of 36 ^o^C and 5000 kPa, the H_2_ conversion is 76% and the H_2_O_2_ selectivity is 82%. The unreacted H_2_ is recovered and recycled back to the reactor.

The produced hydrogen peroxide is used to oxidize the ethylene to ethylene oxide and water as presented in Eq. 4^40–42^.

4 $$\:{\text{H}}_{2}{\text{O}}_{2}+{\text{C}}_{2}{\text{H}}_{4}\:\:\to\:\:\:\:{\text{C}}_{2}{\text{H}}_{4}\text{O} + H_{2}O$$

### Hydration of Ethylene Oxide

There are two types of hydration of ethylene oxide for producing ethylene glycol; direct hydration of EO with water and indirect hydration of EO through intermediate ethylene carbonate to form MEG.

#### Direct hydration of ethylene oxide

**Fig. 3:**
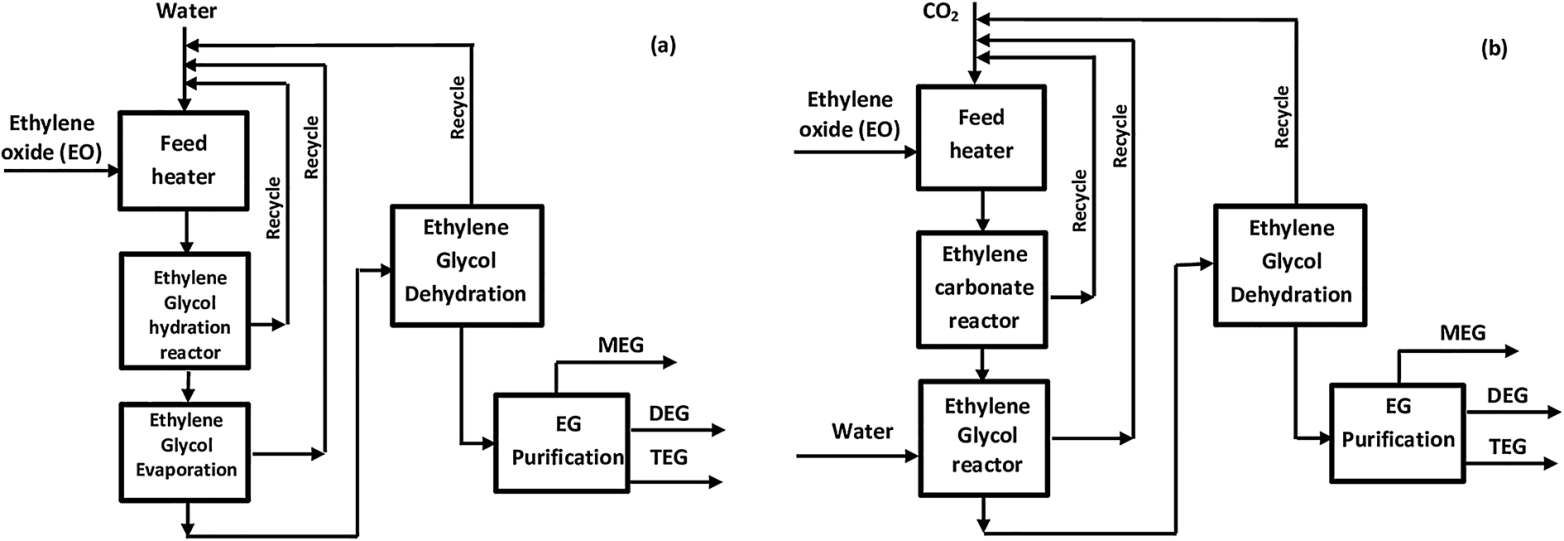
Block diagram of: (**a**) Direct hydration of ethylene oxide; (**b**) Indirect hydration of ethylene oxide through formation of ethylene carbonate intermediate.

Figure (3,a) presents a block diagram of the direct hydration of ethylene oxide. In the feed heater, EO - water mixture is heated from 20 °C to 270 °C and sent to the EG reactor. Glycol reactor feed with 10:1 mol ratio of water: EO is designed to produce the maximum amount of mono ethylene glycol (MEG) (Eq. 5).

5 $$\:{\text{C}}_{2}{\text{H}}_{4}\text{O}+{\text{H}}_{2}\text{O}\:\:\to\:\:\:\:{\text{C}}_{2}{\text{H}}_{6}{\text{O}}_{2}$$

Side reactions may take place, resulting in Di-ethylene glycol (DEG) (Eq. 6), Tri-ethylene glycol (TEG) (Eq. 7), and higher glycol grades. To minimize the occurrence of such unwanted reactions, greater mole ratio of water could be used. Unfortunately, it is difficult to prevent the formation of such homologs since their formation rate is faster than the rate of forming MEG. The effluent of the reactor is coming out at 199 °C.

6 $$\:{\text{C}}_{2}{\text{H}}_{4}\text{O}+{\text{C}}_{2}{\text{H}}_{6}{\text{O}}_{2}\:\to\:\:\:\:{\text{C}}_{4}{\text{H}}_{10}{\text{O}}_{3}$$

7 $$\:{\text{C}}_{2}{\text{H}}_{4}\text{O}+{\text{C}}_{4}{\text{H}}_{10}{\text{O}}_{4}\:\to\:\:\:\:\:\:{\text{C}}_{6}{\text{H}}_{14}{\text{O}}_{5}$$

The EG reactor effluent is sent to series of evaporators for removal of water (Ethylene Glycol Dehydration) depending on the capacity of water to be removed. High-pressure steam provides heat in the first evaporator. The pressure in both evaporators is gradually reduced to minimize MEG loss. A large amount of water is removed from the evaporator train. Two columns operated under vacuum are used for MEG purification- dehydration column and MEG column. The separation process is energy-intensive due to large quantities of water present in the reactor effluent^49^.

#### Indirect hydration process of ethylene oxide to MEG through ethylene carbonate as intermediate

In the direct hydration process of ethylene oxide, the ethylene glycol is produced by the hydration of ethylene oxide in absence of a catalyst. Though, in the hydration process, many other by-products such as di and tri-ethylene glycols are formed. To decrease such side reactions for the production of high yield of Mono-ethylene glycol, a large amount of water is needed, about 10 to 25 moles of water for each one mole of ethylene oxide. This results in the requirement of more energy for the efficient removal of excess water in the distillation step. This method results in a yield of about 90% of ethylene glycol, which is not adequate^3^. For that reason, mono ethylene glycol production by direct hydration of ethylene oxide is preferred in the case of large-scale industrial production of MEG. However, direct hydration process has many drawbacks: the requirement of high mole ratio of H_2_O to EO, the excessive process duration, the lower selectivity of MEG and higher consumption of used energy for the evaporating the water of the products^50,51^. A promising alternative for the production of ethylene glycol is the usage of the ethylene carbonate (1, 3- dioxolan-2-one) as an intermediate compound (Fig. 3b). This compound could be synthesized in high conversion efficiency (98%) by the reaction of ethylene oxide with carbon dioxide; it could be then hydrolyzed to produce a high yield of ethylene glycol. The required molar quantity of water is doubled in that case^27,29,51,52^.

Catalysts used in this reaction could be quaternary ammonium and phosphonium salts. Although this process has some drawbacks such as the method of product separation and catalyst feedback, this technique still considered as the most promising for industrial-scale application for the selective synthesis of ethylene glycol from ethylene oxide^3^. Catalysts used in such reaction could be molybdenum compound, tungsten compound, alkali metal carbonate or quaternary phosphonium halide. Ionic liquid of silica-supported [HCO_3_] is considered as an effective and reused catalyst for hydrolysis of ethylene carbonate.

Scientific Design-Shell Chemical Company and Dow Chemical Company are the main licensors of ethylene oxide process technology. Relied on the proprietary catalyst, Shell Chemical Company introduced two versions of EO/EG technology. The first version is the Shell MASTER Process, and the second version is the Shell OMEGA process.

The catalytic MEG (Shell OMEGA) process has many advantages, which are the low water/EO ratio and high selectivity of MEG; the ratio of water/EO is close to stoichiometry. As a result, the purification section will be more simple, which consequently reduce the required energy consumption, used in water separation, as well as the constructions cost. In addition, this process is high selective for MEG; this accordingly leads to the need for only purification unit and substantially no facility for handling by-product is required. Moreover, the plant operation will be energy-efficient and extremely stable.

In this work, a catalytic MEG process (Shell OMEGA) was recommended and studied for ethylene glycol production.

The process of ethylene glycol synthesis using catalytic hydrolysis of ethylene carbonate consists of two main reactions; carbonation and hydrolysis^52^.

The carbonation reaction is the carbonation of ethylene oxide to produce ethylene carbonates as presented in Eq. 8. The hydrolysis reaction is the hydrolysis of ethylene carbonates to produce ethylene glycol and carbon dioxide (Eq. 9).

8 $$\it \it \it \it \it EO+CO_2{\rightarrow}EC$$

9 $$EC+H_2O{\rightarrow}EG+CO_2$$

The reaction of EO with carbon dioxide is catalyzed by a wide range of compounds.

In this process, carbon dioxide is used as a raw material for the reaction in the carbonation step and is produced as a by-product with the final product in the hydrolysis step. The produced carbon dioxide from hydrolysis reaction is separated, compressed and recycled to the carbonation input; therefore, only small amounts of carbon dioxide will be needed to compensate for losses caused by the system^27,52^.

In this work, modeling of direct and indirect hydration of ethylene oxide was performed. A comparison between direct and indirect hydration was made by performing a quantitative analysis of their economic and environmental impact.

## Results and discussion

Simulation in this work has been developed to produce 600 thousand tons per year of mono-ethylene glycol (MEG), starting from ethylene with a flowrate of 382 thousand tons per year. In this work, HYSYS software version 11 was used as a simulator for producing MEG applying the considered technologies. The thermodynamic properties of all streams were calculated using the glycol package. The cost estimations and design specifications for various equipment such as heat exchangers and distillation columns require mass and energy information, which can be determined by applying the HYSIS software. Moreover, the energy-efficient design for strippers and distillation columns can be achieved by applying the optimization and tray sizing technique embedded in this software. For the studied technologies with low capital costs compared to other unit operations, the catalyst synthesis and regeneration section are neglected. The solid property estimator technique embedded in HYSYS can be used to estimate the physical properties of the catalysts and other solids used in EO production and their interaction with other components of the reaction mixture. The fluid package or equations selected for modeling is the glycol package. Thermodynamic properties such as compressibility, activity, volume, and fugacity can be calculated by employing these equations.

### Liquid phase ethylene oxidation process

Oxidation of ethylene with hydrogen peroxide (H_2_O_2_) in a selective liquid-phase is applied to produce EO in the presence of a homogeneous methyl-trioxorhenium (MTO) catalyst. The liquid-phase reaction mixture consists of methanol, water, hydrogen peroxide (oxidant), and MTO, and a promoter such as a pyridine N-oxide is mixed with compressed ethylene gas (maintained at 4000–5000 kPa) with in the temperature range of 20–40 °C. It was noted that t-butyl alcohol can replace methanol as a solvent. In this liquid phase reaction, EO is selectively produced in the presence of the MTO catalyst which transfers an oxygen atom from H_2_O_2_ to ethylene. The conservation of the feedstock and reduction of the carbon footprint can be obtained by burning elimination^4,40–42^.

**Fig. 4:**
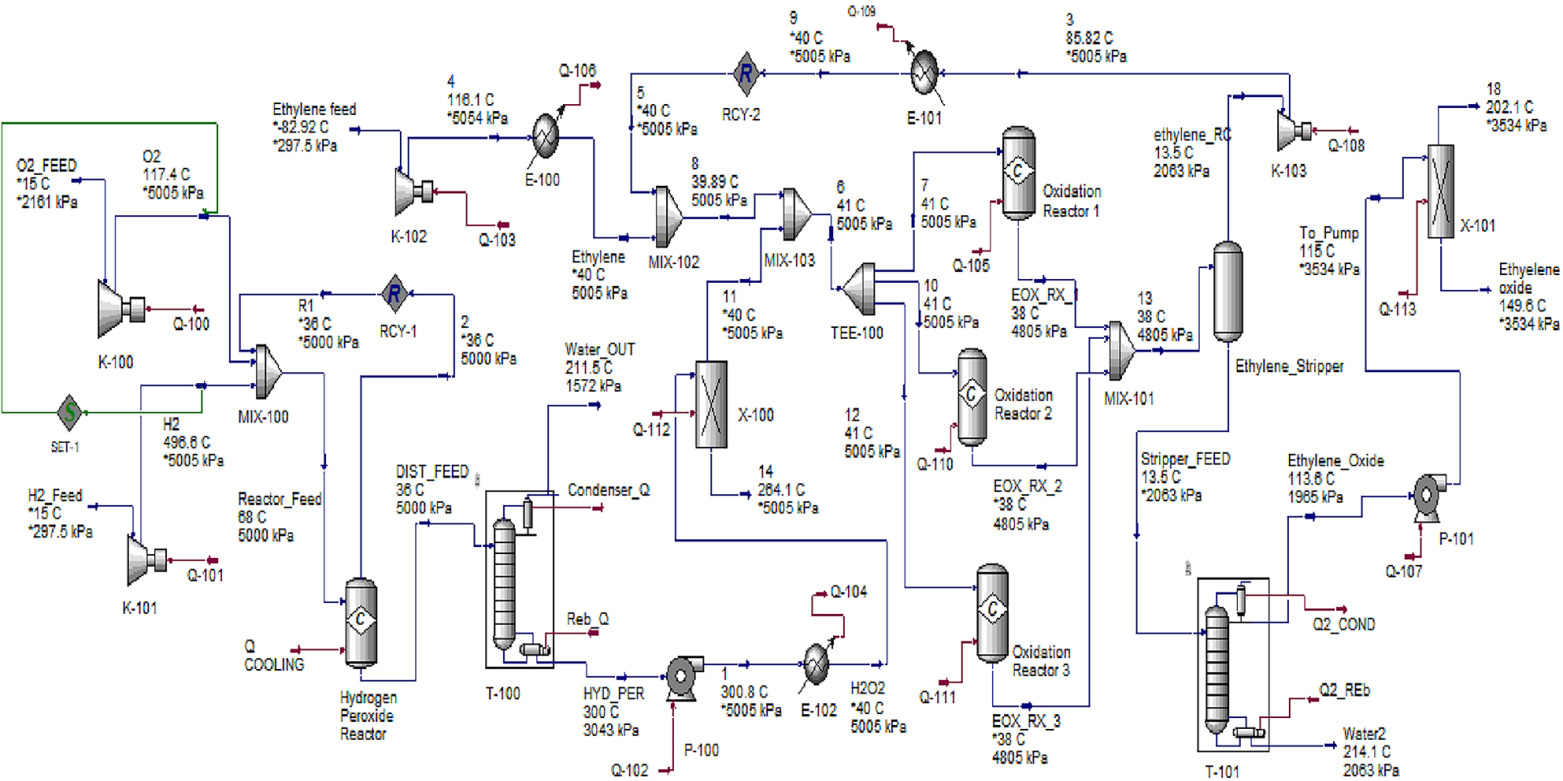
Simulated plant for EO production via ethylene oxidation in the liquid phase.

The process simulation flowsheet of ethylene oxidation in the liquid phase is shown in Fig. 4. It is divided into five sections as follows: (a) H_2_O_2_ preparing reactor (Hydrogen Peroxide Reactor); (b) H_2_O_2_/water separation (T-100 and X-100); (c) liquid-phase oxidation reactors (Oxidation Reactor 1, Oxidation Reactor 2, and Oxidation Reactor 3); (d) ethylene stripper (Ethylene_Stripper) (E) ethylene oxide purification (T-101, and X-101)). The process consists of two production steps the production of H_2_O_2_ and the oxidation of ethylene to EO using H_2_O_2_.

As a second step, ethylene gas is compressed to 5 MPa and converted to the liquid phase. It is then mixed with recycled ethylene. The mixed stream (stream 8) is co-fed with the oxidant H_2_O_2_ to the liquid phase oxidation reactor. The reactor is simulated as a conversion reactor with 98% EO yield and the rest is water. At the operating temperature of 20–40 °C, the vapor phase is devoid of oxygen due to the high stability of H_2_O_2_. At the operating pressure, the obtained EO remains dissolved in the liquid phase. This is useful because EO is flammable in the gas phase. Therefore, for making the process inherently safe, it is required to remove O_2_ and EO from the vapor phase^48^.

It should be noticed that it is not required to deploy inert gases such as N_2_ and AR from the feed stream due to the absence of oxygen in the gas phase reactor. The unreacted ethylene is recovered from the effluent of the oxidation reactor by reducing the pressure from the reactor pressure of 5 MPa to 2 MPa through an ethylene stripper. The recovered ethylene is compressed through the recycled gas compressor (K-103) and is recycled back to the reactor.

For EO purification, EO along with water and carbon dioxide is fed to a distillation column (T-101), where ethylene oxide is recovered from the top of the column. A column boiler duty of 2.75 MW is applied via using HP steam to separate the EO. EO is pumped by the glycol reactor feed pump (P-101) and sent to the EO splitter (X-101). The simulation results of the inlet and outlet streams of the liquid oxidation process are shown in Table 2.

**Table 2:** Simulation results for EO production in the liquid phase as presented in the literature^4,40–42^.

Stream	O2_FEED	H2_Feed	Ethylene feed	Reactor Feed	R1	DIST_FEED	11
Mass flow rate, tone/year	4.362e+05	2.768e+04	3.821e+05	6.405e+06	5,941,000	419,900	3.619e+05
Molar flow, kgmole/h	1556	1556	1555	24,310	21,200	1556	1214
Composition (mole fraction)							
Hydrogen	0.0	0.9991	0.0	0.0639	0.0	0.0	0.0
Oxygen	1.0	0.0	0.0	0.9356	0.9995	0.0001	0.0
Ethylene Oxide	0.0	0.0	0.0	0.0	0.0	0.0	0.0
H_2_O	0.0	0.0009	0.0	0.0004	0.0003	0.2007	0.0
CO_2_	0.0	0.0	0.0	0.0	0.0	0.0	0.0
H_2_O_2_	0.0	0.0	0.0	0.0001	0.0001	0.7992	1.0
Ethylene	0.0	0.0	1.0	0.0	0.0	0.0	0.0

Stream	HYD_PER	7	EOX_RX_1	EOX_RX_2	EOX_RX_3	Stripper_FEED	8
Mass flow rate, tone/year	3.660e+05	3.174e+05	2.843e+05	2.843e+05	2.930e+05	6.437e+05	5.999e+05
Molar flow, kgmole/h	1240	1193	1066	1066	1099	2386	2400
Composition (mole fraction)							
Hydrogen	0.0	0.0	0.0	0.0	0.0	0.0	0.0
Oxygen	0.0	0.0	0.0	0.0	0.0	0.0	0.0
Ethylene oxide	0.0	0.0128	0.3733	0.3733	0.3733	0.4862	0.0193
H_2_O	0.0209	0.0002	0.3758	0.3758	0.3758	0.5089	0.0002
CO_2_	0.0	0.0073	0.0116	0.0116	0.0116	0.0048	0.0110
H_2_O_2_	0.9791	0.3360	0.0	0.0	0.0	0.0	0.0
Ethylene	0.0	0.6437	0.2393	0.2393	0.2393	0.0001	0.9695

Stream	Ethylene_Oxide	12	10	ethylene_RC	18	Ethylene oxide	14
Mass flow rate, tone/year	4.540e+005	3.270e+05	3.174e+05	2.180e+05	6361	4.476e+05	4100
Molar flow, kgmole/h	1183	1229	1193	845.9	23.60	1160	25.98
Composition							
Hydrogen	0.0	0.0	0.0	0.0	0.0	0.0	0.0
Oxygen	0.0	0.0	0.0	0.0	0.0	0.0	0.0
Ethylene Oxide	0.9801	0.0128	0.0128	0.0548	0.0	1.0	0.0
H_2_O	0.0100	0.0002	0.0002	0.0007	0.5021	0.0	1.0
CO_2_	0.0097	0.0073	0.0073	0.0306	0.4860	0.0	0.0
H_2_O_2_	0.000	0.3360	0.3360	0.0	0.0	0.0	0.0
Ethylene	0.0002	0.6437	0.6437	0.9139	0.0119	0.0	0.0

### MEG production via direct EO hydration process

**Fig. 5:**
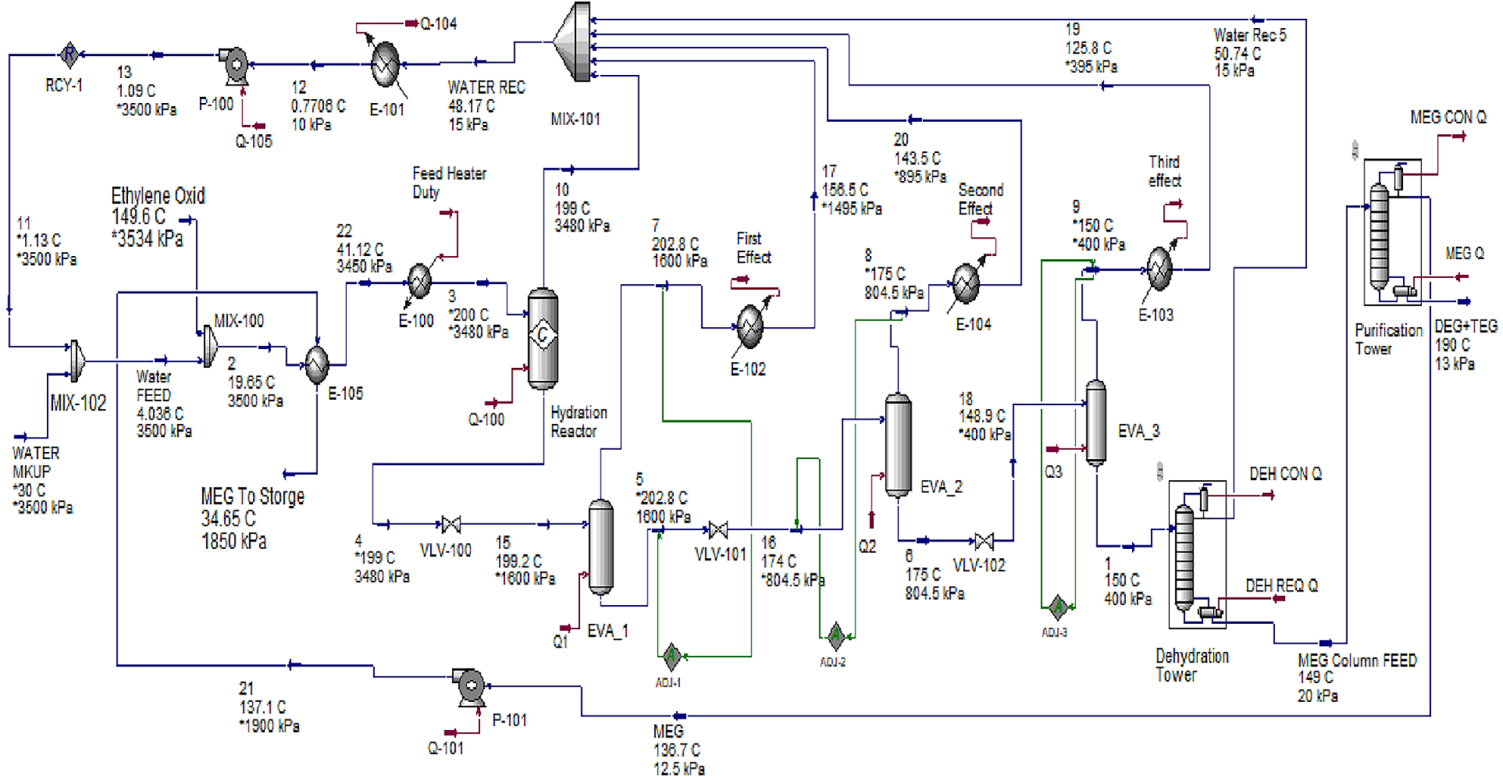
Simulated plant for MEG production via direct hydration of ethylene oxide.

The conventional direct hydration process of EO to produce MEG was simulated based on data and assumptions listed in Table 1 and the resulting process flowsheet is presented in Fig. 5.

Along with ethylene oxide, recycled water is heated to the reaction temperature through Feed/Product heat exchanger (E-105) and feed heater (E-100) with a duty of 4.446 MW then co-fed into the hydration reactor. The hydrolysis reaction of EO to form EG is carried out in a plug flow reactor. The Reactor is 77.8 m long and 0.55 m in diameter. The ethylene oxide hydration reaction is endothermic and sustained at 199 °C and pressure of 3480 kPa with 3 min as residence time. The product distribution from reactor is 88.03 mol% H_2_O, 1.55 mol% EO, 10.24 mol% MEG, 0.1 mol% DEG, and 0.08% TEG. This distribution is controlled by EO and cycle water ratio. To get this distribution, the cycle water to the EO molar ratio is kept as 10:1.

The outlet stream from the EG reactor contains MEG, DEG, TEG, EO, and water. This stream is introduced to three evaporators with total energy consumption of 17.8 MW. Approximately 75% of water is removed in this section using re-boiling and then introduced to the dehydration column. Only the first evaporator uses external steam while the second and third evaporator uses the steam generated in the first and second evaporator respectively. The dehydration column is operated under a vacuum of 85 mm Hg to remove the rest of the water. This separation process is energy-intensive due to the large quantities of water present in the reactor effluent. The simulation results of direct hydration of EO to produce MEG are presented in Table 3.

The glycol purification MEG column is used to separate MEG and DEG + TEG. This column is operated under vacuum to avoid the thermal degradation of MEG because the vacuum leads to operation below the maximum allowable temperature of 170 °C. The bottom of the dehydrator is fed to the MEG column, where MEG is separated from high grades and withdrawn as a side stream.

To control the undesired reactions, ethylene glycol is typically produced in the liquid phase at elevated temperature of 199 °C and pressure of 3480 kPa. The entire process flow can be quite complex and involves not only the reactor, but also three eliminators or steam generators to eliminate any excess water in the reaction mixture, as well as a series of distillation columns to remove any mixture of di- and tri-ethylene glycols from the final commercial product of ethylene glycol. The MEG is cooled with chilled water^53^ in heat exchanger E-105 and sent to the MEG storage Tank.

**Table 3:** Simulation results for producing MEG from direct hydration of EO^24,26,27,29,52^.

Stream	WATER MKUP	11	Ethylene Oxid	21	10	4	5	6
Mass flow rate, tonne/year	1.783e+05	1.602e+06	4.477e+05	6.042e+05	0.0	2.228e+06	2.08e+06	1.874e+06
Molar flow, kgmole/h	1130	9870	1160	1113	0.0	1.10e+004	1.019e+004	8987
Composition (mole fraction)								
Oxygen	0.0	0.0	0.0	0.0	0.0	0.0	0.0	0.0
Ethylene	0.0	0.0	0.0	0.0	0.0	0.0	0.0	0.0
H_2_O	1.0	0.9812	0.0	0.0023	0.8803	0.8803	0.8790	0.8706
EO	0.0	0.0173	1.0	0.0	0.0155	0.0155	0.0089	0.0030
CO_2_	0.0	0.0	0.0	0.0	0.0	0.0	0.0	0.0
MEG	0.0	0.0015	0.0	0.9977	0.1024	0.1024	0.1101	0.1241
DEG	0.0	0.0	0.0	0.0	0.0010	0.0010	0.0011	0.0012
TEG	0.0	0.0	0.0	0.0	0.0008	0.0008	0.0009	0.0010

Stream	1	WATER REC	MEG Column FEED	DEG+TEG	MEG	MEG To Storage		
Mass flow rate, tonne/year	1.701e+06	1.602e+06	6.264e+05	22,270	6.042e+05	6.042e+05		
Molar flow, kgmole/h	7935	9867	1133	20.43	1113	1113		
Composition (mole fraction)								
Oxygen	0.0	0.0	0.0	0.0	0.0	0.0		
Ethylene	0.0	0.0	0.0	0.0	0.0	0.0		
H_2_O	0.8567	0.9812	0.0	0.0023	0.0023	0.0023		
EO	0.0007	0.0173	0.0	0.0	0.0	0.0		
CO_2_	0.0	0.0	0.0	0.0	0.0	0.0		
MEG	0.1400	0.0015	0.9802	0.0231	0.9977	0.9977		
DEG	0.0014	0.0	0.0097	0.5373	0.0	0.0		
TEG	0.0011	0.0	0.0079	0.4396	0.0	0.0		

### MEG production via indirect EO hydration process

**Fig. 6:**
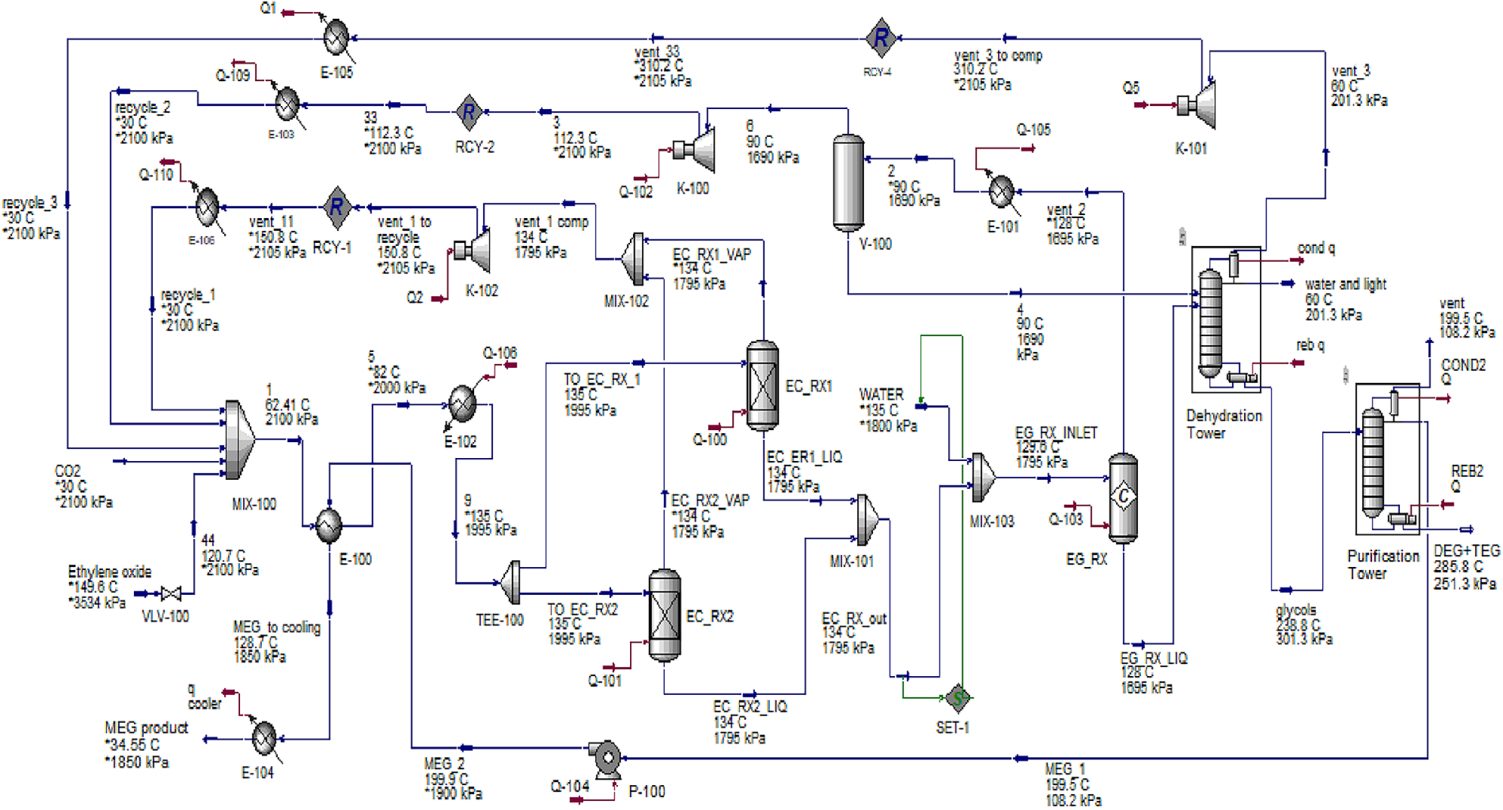
Simulated plant for MEG production through ethylene carbonates formation.

The process simulation flow sheet of MEG production by hydration of EO through ethylene carbonate (EC) process is shown in Fig. 6. The simulation assumptions, equipment characteristics as well as the molar flow rate and composition of the inlet and outlet streams for this process are listed in Table 4. The selective ethylene oxide hydration through EC as an intermediate process could be represented by three sections as illustrated in Fig. 6. The CO_2_ and EO with recycled gases are co-fed to Feed/Product heat exchanger (E-100) then to reactor feed heater (E-102) to be heated to 135 °C, then co-fed to the EC reactors (EC_RX1 and EC_RX2). The reactors were simulated as conversion reactors with a 98% EC yield. Because the reaction is highly exothermic, the absorbed heat could be used for LP steam generation. The produced EC is mixed with water and fed to the glycol reactor (EG-RX). The reaction temperature is maintained at 128 °C by applying cooling water, the reactor is simulated as a conversion reactor with 90% MEG yield. Unreacted ethylene oxide and carbon dioxide gases are recycled back to the EC reactors after compression.

**Table 4:** Simulation results for indirect hydration of EO through the EC process^24,26,27,29,52^.

Stream	CO_2_	Ethylene oxide	MEG_2	TO_EC_RX_1	EC_RX1_VAP	EC_ER1_LIQ	EG_RX_INLET
Mass flow rate, tonne/year	1461	4.477e+05	6.261e+05	5.318e+05	6.537e+04	4.664e+05	1.133e+06
Molar flow, kgmole/h	3.790	1160	1152	1379	165.5	634.2	2537
Composition							
CO_2_	1.0	0.0	0.0	0.5628	0.9471	0.0634	0.0317
MEG	0.0	0.0	0.9984	0.0003	0.0007	0.0007	0.0004
Ethylene Oxide	0.0	1.0	0.0	0.4284	0.0243	0.0123	0.0061
H_2_O	0.0	0.0	0.0009	0.0053	0.0028	0.0109	0.5054
TEG	0.0	0.0	0.0	0.0	0.0	0.0	0.0
DEG	0.0	0.0	0.0	0.0	0.0	0.0	0.0
EC	0.0	0.0	0.0007	0.0032	0.0258	0.9128	0.4564

Stream	6	9	EG_RX_LIQ	EC_RX2_VAP	EC_RX2_LIQ	recycle_1	water
Mass flow rate, tone/year	4.664e+05	1.064e+06	6.627e+05	6.537e+04	4.664e+05	1.307e+05	2.002e+005
Molar flow, kgmole/h	1215	2757	1312	165.5	634.2	331.1	1268
Composition (mole fraction)							
CO_2_	0.9838	0.5628	0.0295	0.9471	0.0634	0.9471	0.0
MEG	0.0007	0.0003	0.8740	0.0	0.0007	0.00	0.0
Ethylene Oxide	0.0077	0.4284	0.0034	0.0243	0.0123	0.0243	0.0
H_2_O	0.0076	0.0053	0.0891	0.0028	0.0109	0.0028	1.0
TEG	0.0	0.0	0.0001	0.0	0.0	0.0	0.0
DEG	0.0	0.0	0.0011	0.0	0.0	0.0	0.0
EC	0.0001	0.0032	0.0028	0.0258	0.9128	0.0258	0.0

Stream	recycle_2	water and light	glycols	DEG+TEG	recycle_3	MEG 1	MEG product
Mass flow rate, tone/year	4.664e+05	2.166e+04	6.276e+05	1532	1.719e+04	6.261e+05	6.261e+05
Molar flow, kgmole/h	1215	119.4	1153	1.601	47.25	1152	1152
Composition (mole fraction)							
CO_2_	0.9838	0.0007	0.0	0.0	0.8236	0.0	0.0
MEG	0.0007	0.0193	0.9971	0.0272	0.0001	0.9984	0.9984
Ethylene Oxide	0.0077	0.0059	0.0	0.0	0.0802	0.0	0.0
H_2_O	0.0076	0.9502	0.0008	0.0	0.0958	0.0009	0.0009
TEG	0.0	0.0	0.0001	0.0973	0.0	0.0	0.0
DEG	0.0	0.0	0.0012	0.8755	0.0	0.0	0.0
EC	0.0001	0.0239	0.0007	0.0	0.0004	0.0007	0.0007

The outlet of the MEG reactor is fed to the dehydration tower at 128 °C to separate water from the MEG product. The tower reboiler duty of 10.763 MW is applied to separate water from the top of the distillation. The undesired di-ethylene-glycol (DEG) and tri-ethylene-glycol (TEG) which are produced as by-products are separated from the desired MEG in the purification column. The purification column with a reboiler duty of 30.66 MW given by using high-pressure steam is used to separate DEG and TEG from MEG.

The fractionation is so simple without consumption of energy in the case of MEG production through the EC process compared to the direct hydration process. The flow rate of the DEG and TEG is 1532 tonne/year, which is very low compared to their flowrate in the direct hydration process (22,270 tones/year). The produced MEG is withdrawn from the distillation column at 199.5 ^o^C and sent to the feed/product heat exchanger to heat the fresh feed. MEG out of this heat exchanger at 128.7 °C is sent to the cooler to exit at 34.55 °C then to storage.

### Model validation

In this work, simulations of the presented processes were performed based on operating data published in the literature and patents for oxidation of ethylene to ethylene oxide^4,40–42^ and for direct and indirect hydration of EO to MEG^24,26,27,29,52^. The physical properties of the final monoethylene glycol obtained from simulation models of direct and indirect ethylene oxide hydration processes were validated based on experimental data from previous studies^4,54–57^. Table 5 compares the physical properties of MEG obtained from simulations of the presented hydration processes and those of MEG reported in the literature. The physical properties of MEG obtained from models of hydration processes except for viscosity agreed with the physical properties reported for MEG in previous studies with a minimum error of 0.0206% and a maximum error of 2.3%. Regarding viscosity, the presented model showed an error of 17.3%. Although the viscosity error is large, considering that a difference lies in the uncertainty in the prediction from simulations, and the measured viscosity is less than 1.0 Pa.s, an error of 17.3% is considered an acceptable result. From the results, we can conclude that the presented model is able to produce a MEG product with physical properties close to the standard ones.

**Table 5:** Comparison of the physical properties of MEG produced from simulation and that reported in the literature^4,55–58^.

Properties item	MEG produced from simulation (EO/MEG, EC/MEG)	MEG from literature studies	Error, %
Boiling point at 101.3 kPa	197.5 ^o^C	197.6 ^o^C	0.050
Molecular weight	62.07 g/mole	62.1 g/mole	0.048
Normal freezing point	-13.3 ^o^C	-13.0 ^o^C	2.30
Density at 20 ^o^C	1114 kg/m^3^	1113.5 kg/m^3^	0.045
Heat of vaporization at 101.3 kPa	52.87 kJ/mole	53.2 kJ/mole	0.620
Surface tension at 25 ^o^C	0.04797 N/m	0.048 N/m	0.062
Viscosity at 20 ^o^C	16.37 *10^-3^ Pa.s	19.8 *10^-3^ Pa.s	17.3
Specific gravity (20/20°C)	1.113	1.1153	0.206

### Economic assessment

#### Economic study of ethylene oxidation for producing ethylene oxide

The estimated total capital investments for the liquid phase processes for ethylene oxidation are approximately 31 million dollars.

EO production plant includes two pumps. The estimated pump cost for the liquid oxidation processes are approximately 0.21 million dollars. There are four compressors in the liquid oxidation process, two of them are used to compress the O_2_ and H_2_ before the hydrogen peroxide reactor, one compressor is used for ethylene compression, and the last compressor is the recycle gas compressor. The ethylene and recycle gas compressors’ cost for the liquid phase oxidation processes is 5.2 million dollars.

The cost of distillation columns in the liquid phase oxidation processes is approximately 12 million dollars. The higher cost of the distillation columns can be attributed to the stainless steel (SS-304) alloy that has been chosen as a material of construction for liquid oxidation columns to resist the metal decomposition of H_2_O_2_. The estimated costs of heat exchangers, and vessels and separators are approximately 1.2 and 0.018 million dollars, respectively. The total operating cost includes the following items^44^:

- The raw material cost: the total raw material cost for the simulated model per year.
- The operating labor and maintenance: the cost of operators and supervisor plus the maintenance cost.
- The operating charges: 25% of the percent of operating labor costs.
- Plant overhead: 50% of the operating labor and maintenance costs.
- Utilities cost: the total utility consumption cost expressed in $ per year.

The cost of ethylene as the main raw material in the oxidation process was taken as $0.7 per kg, while the cost of the oxidant (H_2_O_2_) was $0.6/kg. It should be noticed that all costs of various raw materials and products used in this research work are taken from the chemical prices website^49^. The total raw material cost for the liquid phase oxidation processes is MM$ 316.868. The higher raw material cost can be attributed to the consumption of H_2_ for producing hydrogen peroxide.

The total operating labor includes the operators, supervisors, maintenance labor, and supplies labor. The assumption has been made are fixed for all the simulation models in this study. The maintenance cost is a function of the equipment types and numbers used in each simulation. The estimated yearly maintenance cost is assumed to be 5–9% of total capital cost for average processes with normal operating conditions. Also, the number of operators depends on the plant size and the number of equipment in each simulation case. The operating labor has been calculated to be 7 operators with one supervisor. The labor cost was taken as $20 per hour for the operator and $35 per hour for the supervisor^48^. Accordingly, the estimated annual total labor costs is MM $ 1.47.

One of the most important utilities is saturated steam. The liquid phase process operates at near-ambient temperatures, the reaction exotherms are not enough for producing process steam that can be used in distillations and electricity generation. As reviewed later the utility costs and consumption are taken from the Aspen HYSYS utility manager version 11. The annual utility costs, consumption, and generation are estimated to be MM$ 20.9. Table 6 illustrates all types of operating costs of oxidation of ethylene to EO in the liquid oxidation.

The total product sales is calculated based on $ 0.9 per kg of ethylene oxide. The estimated total product sales is MM$ 403.162 per year.

The payback period refers to the time required to recover the capital cost of a plant. Regarding Table 6, it is clear that liquid phase process has a low payback period of 0.813 years.

**Table 6:** Total economic study of liquid oxidation of ethylene to EO.

Items	
Total Raw Materials Cost [USD/Year]	316,868,000
Operating labor cost, $/year	1,470,000
Maintenance (5-9% total capital cost), $/year	2,200,464
Utilities cost, $/year	20,945,900
Operating charges cost (percent of operating labor costs), (25% operating labor), $/year	367,500
Plant overhead cost (percent of operating labor and maintenance costs), $/year (50% (operating labor + maintenance)	1,835,232
Subtotal operating costs, $/year	343,687,096
General expenses costs (percent of subtotal operating costs), $/year (With percent 8%)	27,494,968
Total product cost, $/year	371,182,064
Total Product Sales, $/year	403,162,000
Profit (Product sales –Product cost), $/year	31,979,936
Net profit (Profit – taxes) (With 20% income taxes), $/year	25,583,949.06
Total Capital Cost, $	31,435,200
Return on investment, yr^-1^	1.228
Payback period, yr	0.813

#### Economic assessment of the two considered hydration processes for producing MEG

The estimated total capital investment for the direct hydration ethylene oxide process (EO-MEG) and indirect hydration through ethylene carbonate (EC-MEG) process used to produce MEG are both approximately the same amount of $70 million and the difference is within the uncertainty expected from the preliminary economic analysis (± 25%). The indirect hydration process includes an additional reaction section equipped with utilities, feed preparation, and product separation sections used to produce EC. The cost of reactors for the EC-MEG process is approximately MM$ 58, which is much higher than the direct hydration process cost of MM$ 25. Despite the cost of these reactors is higher in the indirect EC-MEG hydration process, the total capital cost for the two hydration processes are equal (MM$ 70). The capital cost of the direct hydration EO-MEG process is high due to the presence of a water separation section which includes 3 evaporators and two distillation columns. The three evaporators are used to remove water from EO which enters the evaporators with a molar ratio of 10:1 for H_2_O: EO. This purified EO is hydrated to achieve a 96 wt% yield of MEG with limited amounts by-products. After water evaporation, the two distillation columns are used to remove the rest of the water and to separate the desired MEG from DEG and TEG by-products. The total cost of these two columns is 31 million dollars, compared to 9 million dollars for the two distillation columns used in the EC-MEG process.

Unlike direct hydration, the EC-MEG process contains a simple separation section where a dehydration column is used to remove the produced water and a purification column to increase the purity of MEG. Table 7. presents a summary of the equipment cost for the two considered hydration processes used to produce MEG.

Regarding Table 7., the pumps’ costs in the EC-MEG and EO-MEG processes are approximately 0.090 and 0.353 million dollars, respectively. Besides, the cost of the compressors for the EC-MEG is 1.77 million dollars, while there are no compressors used in the EO-MEG process. For the EO-MEG, process, unlike the EC-MEG process, all reactions and separations processes have occurred in the liquid phase without the formation of any vapor streams that could require to be compressed through compressors. The evaporators with steam ejectors used in the EO-MEG process cost 8.6 million dollars while for the EC-MEG technique, no evaporators are existed due to the lower mole ratio of EO to H_2_O.

The estimated heat exchangers costs in the EC-MEG process are 0.916 million dollars compared to 4.1 million dollars for the EO-MEG process. The estimated costs for vessels and separators are approximately 0.355 and 0.268 million dollars for the EO-MEG and EC-MEG techniques respectively.

**Table 7:** Equipment cost for the two considered hydration processes.

Equipment	Cost of direct hydration of Ethylene oxide for producing MEG ($)	Indirect hydration of ethylene oxide for producing MEG by forming Ethylene carbonate, ($)
Reactors	25,212,300	58,187,660
Columns	31,433,000	9,074,930
Compressors	-	1,774,479
Heat Exchangers	4,143,600	916,400
Pumps	352,800	89,846
Vessels and Separators	354,800	267,968
Evaporators and ejectors	8,635,600	-
Total	70,132,100	70,311,282

For the two investigated hydration processes, 1160 kgmole/h of EO has been used to produce 600 thousand tonne annually of MEG, which is the main target of the understudy project.

The water separation section in the EO-MEG process consists of three evaporators and one distillation column (Dehydrator). This section consumes 219.258 MW for heating purposes for producing 600 thousand tonne of MEG. However, due to the small amount of water needed for the EC-MEG hydration process, it requires only one dehydration column for water separation. Thus, water separation for this process consumes 10.763 MW and 3.115 MW as reboiler and condenser duties respectively. The weight% of DEG and TEG in the produced glycol mixture is 3.56 wt% for the EO-MEG process compared to 0.244 wt% in the case of the EC-MEG approach. To separate DEG and TEG from MEG, a distillation column is needed. Therefore, the glycols separation section consumes 20.87 MW and 21.51 MW as reboilers and condensers duties applied on the distillation column for the EO-MEG process compared to 30.7 MW for reboilers and 33.5 MW for condensers in the case of EC-MEG technology. The summary of the energy consumption for both investigated hydration processes is addressed in Table 8. It is noted that the energy consumption in the direct hydration (323 MW) is higher than the energy consumption in EC hydration (54.7 MW). That is because the high energy required for the condenser and the reboiler of the dehydrator tower to recover the water from the produced ethylene glycol.

**Table 8:** Energy streams of direct and indirect hydration of EO to produce MEG.

EO-MEG process										
Name	Feed Heater Duty	Q1	Q3	DEH CON Q	DEH REQ Q	MEG CON Q	MEG Q	Q-104		
Heat flow, kW	44462.57	8879.99	5037.75	107392.75	94108.24	21513.56	20870.47	16645.27		
Name	Q-105	First Effect	Q2	Third Effect	Second Effect	Q-100	Q-101			
Heat flow, kW	231.27	8348.11	3839.25	11709.60	12793.01	-32917.29	47.13			
EC-MEG process										
Name	cond q	reb q	COND2 Q	REB2 Q	Q-102	Q-101	q cooler	Q3	Q-109	Q-110
Heat flow, kW	3115.59	10763.39	33537.07	30660.33	285.02	-36097.40	5392.98	285.02	1320.17	657.90
Name	Q-103	Q-104	Q-105	Q-108	Q-100	Q-106	Q1	Q2	Q5	
Heat flow, kW	35597.49	49.07	688.54	1292	-36097.40	2773.94	233.08	63.97	142.60	

The calculated total operating cost for the direct hydration process is approximately MM$ 452 compared to MM$ 416 for the EC-MEG hydration process as listed in Table 9.

**Table 9:** Product costs for the two considered hydration processes.

EO-MEG process	
Total Raw Materials Cost [USD/Year]	403,166,000
Operating labor cost, $/year	1470000
Maintenance Cost, $/year	4,909,247
Utilities cost, $/year	39,045,600
Operating charges cost (percent of operating labor costs), (25% operating labor), $/year	367500
Plant overhead cost (percent of operating labor and maintenance costs), $/year (50% of (operating labor + maintenance)	3,189,624
Subtotal operating costs, $/year	452,147,971
General expenses costs (percent of subtotal operating costs), $/year (With percent 8%)	36,171,838
Total product cost, $/year	488,319,809
EC-MEG process	
Total Raw Materials Cost [USD/Year]	403,473,000
Operating labor cost, $/year	1,470,000
Maintenance Cost, $/year	4,921,790
Utilities cost, $/year	2,381,340
Operating charges cost (percent of operating labor costs), (25% operating labor), $/year	367500
Plant overhead cost (percent of operating labor and maintenance costs), $/year (50% of (operating labor + maintenance)	3,195,895
Subtotal operating costs, $/year	415,809,525
General expenses costs (percent of subtotal operating costs), $/year (With percent 8%)	33,264,762
Total product cost, $/year	449,074,287

The total product sales for both hydration techniques have been calculated based on $1.2 per kg for MEG^58^. These price values have been selected according to the available average market share. The resulted total annual product sales for the EC-MEG hydration process is approximately MM$ 751 compared to MM$ 725 for the direct hydration process. The increase in sales of EC/MEG process products compared to EO/MEG process sales is due to the increase in the MEG flow rate from the EC/MEG process (626,100 tons/year) compared to the MEG flow rate from the EO/MEG processes (604,200 tons/year). The overall economic summary of the two-hydration processes studied is shown in Table 10. As shown in the table, the indirect EC-MEG hydration process has a higher net profit and return on investment and a shorter payback period. Thus we can conclude that the EC/MEG process is economically more preferable compared to the EO-MEG direct hydration technique.

**Table 10:** Total economic study for the two investigated hydration processes.

EO-MEG process	
Item	
Total Capital Cost, $	70,132,100
Total product cost, $/year	488,319,809
Total Product Sales, $/year	725,040,000
Profit (Product sales –Product cost), $/year	236,720,191
Net profit (Profit – taxes) (With 20% income taxes), $/year	189,376,153
Return on investment, yr^-1^	2.723
Payback Period, yr	0.37

EC-MEG process	
Total Capital Cost, $	70,311,282
Total product cost, $/year	449,074,287
Total Product Sales, $/year	751,320,000
Profit (Product sales –Product cost), $/year	302,245,713
Net profit (Profit – taxes) (With 20% income taxes), $/year	241,796,570
Return on investment, yr^-1^	3.44
Payback Period, yr	0.29

### Environmental impact assessment

#### GHG emissions from oxidation of Ethylene to ethylene oxide

The main GHG emissions from the studied processes are CO_2_ emission as determined by the HYSYS software. Table 11 describes the greenhouse gas emissions resulting from the process of oxidation of ethylene to ethylene oxide in the liquid phase using hydrogen peroxide as the oxidizing agent. As shown, the greenhouse gas emissions associated with the oxidation of ethylene to ethylene oxide are 616,039 tons of carbon dioxide equivalent per year. The liquid phase oxidation process produces 4422 tons/year in process emissions (CO_2_ as byproduct). It is assumed that ethylene and hydrogen are produced as raw materials for this process by cracking ethane (ethane is recovered from natural gas) where ethylene is produced by cracking ethane and hydrogen is produced as a by-product in the same process. In this work, the mole fraction of natural gas composition used as feedstock for ethane recovery is methane (0.752), ethane (0.0970), propane (0.064), i-butane (0.0105), n-butane (0.0065), i-pentane (0.003)., n-pentane (0.0015), n-hexane (0.0008), n-heptane (0.0006), water (0.0187), hydrogen sulfide (0.0157), carbon dioxide (0.0287), and nitrogen (0.001). There are some operations carried out on natural gas. These processes are the sweetening process (to remove carbon dioxide causing corrosive carbonic acid in the presence of water and hydrogen sulfide which causes corrosion of pipelines and equipment), the drying process to remove the water content, and gas compression to separate methane, ethane and natural gas liquids. The sweetening process is applied in the presence of methyldiethanolamine (MDEA)^39,40,59,60^. The separated ethane is then cracked into ethylene and hydrogen by steam cracking. 20,000 kgmole/h of natural gas is used to produce 1,555 kgmole/h (409,700 t/y) of ethane. The recovered ethane is cracked into 1555 kgmole/h (382,100 tons/year) of ethylene and 1556 kgmole/h (27,680 tons/year) of hydrogen as a byproduct. The two processes (ethane recovery from natural gas and ethane cracking) are modeled and simulated based on HYSYS data and estimated GHG emissions of 46,911 tons of CO_2_ equivalent per year (GHG emissions from ethane recovery process) and 85,777 from utility emissions from ethane cracking. The main hotspots and their percentage contribution to the total GHG resulting from the ethylene oxidation process to ethylene oxide are shown in Table 11 (column 2). As shown in the table, the carbon dioxide produced as a by-product is 0.7% of total greenhouse gas emissions, and natural gas-based power generation (energy required) for gas compression is responsible for approximately 21.983% of total greenhouse gas emissions that contribute to global warming and climate change. The main hot spots in the oxidation process are natural gas-based energy used for water cooling (59.9%). While greenhouse gas emissions resulting from the production of ethylene as a raw material are about 21.5%. Table 11 also compared the GHG emissions estimated by the Aspen HYSYS software for natural gas power generation and the GHG emissions for natural gas power generation from the literature^40^. It should be noted that the potential power generation emissions predicted by HYSYS are of the same order of magnitude as reported by Ghanta et al.^40^ with predictions being smaller than those reported by Ghanta et al. In addition, Table 11 also compares the GHG emissions from raw material manufacturing processes (the process of extracting ethane from natural gas and cracking ethane into ethylene and hydrogen) from the presented study and the GHG emissions reported for the same raw materials by other studies^40^. The production capacity of the previous study is 400,000 tons of ethylene/year, which is comparable to that used in the simulation of 382,100 tons of ethylene/year. As shown in the table, GHG emissions from raw material manufacturing processes are 132,688 tons of CO_2_-Eq. per year, which is the same order of magnitude as predicted by previous studies (167,000 tons of CO_2_-Eq. per year), with the predicted emissions being lower than reported by Ghanta et al. This difference in prediction may be due to the lower production capacity of the simulation compared to that in the previous study. The environmental impacts that differs by an order of magnitude to be reliable for making conclusion about the relative impact of the competing processes^40^.

**Table 11:** GHG emissions from the oxidation process of 382,100 tons ethylene/yr for producing ethylene oxide.

Item	tons CO_2_ equivalent/year (estimated by HYSYS software based on USEPA^47^)	Comparing to other studies
GHGs emissions from reaction section	4422 (0.7% of total GHG emissions)	
GHGs emissions from utilities		
Cooling water	369,100 (59.9%)	
HP Steam	82,950 (13.5%)	
LP Steam	-	
LP steam Generation	-	
MP Steam	-	
MP Steam Generation		
Refrigerant (propane)	4893 (0.8%)	
Power generation emissions	21,983 (3.6%) (based on 55.89 kg CO_2_ /1000 Mega joule from natural gas [40, 47])	26,455 (based on 67.26 kg CO_2_ equivalent/1000 Mega joule of energy from natural gas)^41^
Total utilities emissions	483,351 (78.46% of total GHG emissions)	
GHG emissions from raw material production process	Ethylene + hydrogen (based on processing of 3.751 million tons/yr natural gas for producing 409,700 tons/yr ethane) + (cracking of recovered ethane for producing 382,100 tons/yr ethylene and 27,680 tons/yr hydrogen as a byproduct)	
	132,688 (21.5%) - 46,911 from recovery process of ethane from natural gas - 85,777 from ethane cracking process	167,000 (based on producing 400,000 tons/y ethylene from natural gas)^41^
Total	616,039	

#### GHG emissions from the direct and indirect hydration of ethylene oxide processes

The GHG emissions of the two hydration processes studied are summarized in Table 12 As shown in the table, the GHG emissions associated with MEG production from direct and indirect hydration are 1,167,628 and 763,901 tons CO_2_ Eq. per year, respectively.

For the reaction section in EO/MEG and EC/MEG operations, there are no process emissions from the reaction section as a result of all venting emissions from the reaction section being recycled to be mixed with the ethylene oxide feed.

Regarding the EO/MEG process, GHG emissions from utilities and raw material production processes are approximately equal in their negative environmental impact. Greenhouse gas emissions resulting from utilities are about 47%, while greenhouse gases resulting from raw material production are about 53%. The increase in utilities emissions (551,589 tons CO_2_ Eq. per year) is due to the natural gas-based energy needed to cool the water used in cooling the recycling streams (14.13% of total GHG emissions), the medium-pressure steam required for the second evaporator EVA_2 (14.73% of total GHG emissions), and the high-pressure steam required for the E-100 feed heater and evaporators EVA_1 and EVA_2 (12.42% of GHG emissions). The higher energy required results from the increased water/EO molar ratio in the reaction section used to reduce the higher grades of ethylene glycol to be formed. In contrast, in the case of the EC/MEG process, the GHG emissions of utility-based natural gas are much lower. It represents about 19.36% of the total greenhouse gas emissions resulting from the process. The reduction in facility emissions compared to the EO/MEG process results from the fact that there is no need for additional water in the reaction section and therefore there is no need to use evaporators. This results in a reduction in steam consumption and an approximately 27.88% reduction (403,727 tons CO_2_ Eq. per year) in total utility GHG emissions compared to the EO/MEG process. On the other hand, the GHG emissions from manufacturing different raw materials are the same in the two processes with the same ethylene oxide as the process feedstock while neglecting the manufacturing emissions from carbon dioxide as another raw material in the EC/MEG process since carbon dioxide can be obtained as a by-product in many processes in petroleum and petrochemical plants.

**Table 12:** GHG emissions from the studied hydration processes.

Item	EO-MEG process	EC-MEG Process
GHGs emissions from reaction section, tons/year	-	-
GHGs emissions from utilities, tons CO_2_ equivalent/year		
Cooling water	1.65E+05 (14.13%)	1.462E+04 (1.91%)
HP Steam	1.45E+05 (12.42%)	-
LP Steam	-	-
LP steam Generation	2.43E+04 (2%)	1.274E+05 (16.68%)
MP Steam	1.72E+05 (14.73%)	4889 (0.64%)
MP Steam Generation	1.54E+04 (1.32%)	-
Refrigerant (propane)	3.03E+04 (2.6%)	
Power	488.6 (0.04%)	953 (0.124%)
Total utilities emissions	551,589 (47.24%)	147,862 (19.36%)
GHG emissions from raw material production process, tons CO_2_ equivalent/year	Ethylene oxide (based on ethylene oxidation)	Ethylene oxide (based on ethylene oxidation )
	616,039 (52.76%)	616,039 (80.64%)
Total	1,167,628	763,901

## Conclusion

Ethylene glycol serves as an anti-freezing liquid and is a crucial raw material for synthetic fibers and resins. In the absence of a catalyst, it is typically formed by the direct hydration of ethylene oxide. The primary drawbacks of this method are an extremely high mole ratio of H_2_O to EO; a longer process flow; reduced MEG selectivity; and a higher energy consumption, with the majority of the energy going toward evaporating the products’ water. An appealing substitute appears to be the selective production of ethylene glycol using the intermediate ethylene carbonate (indirect hydration). With this method, ethylene carbonate can be hydrolyzed selectively to give a large amount of ethylene glycol. Comparative economic and environmental evaluations of direct and indirect hydration processes for MEG production were performed based on a simulation of 392,000 tons of ethylene to produce 600,000 tons of MEG per year.

The simulation and economic assessment results of the two investigated hydration process of EO to produce MEG showed that the direct hydration (referred as EO-MEG) process is an energy-intensive process, due to the high molar ratio of water to EO, which make the separation process is costive and complicated compared to the EC-MEG hydration process. In addition, the EC/MEG process has a higher net profit, higher return on investment, and shorter payback period when compared to the EO/MEG process. Therefore, the indirect EC-MEG hydration process is economically more preferable compared to the direct EO-MEG hydration process.

Regarding the environmental impact assessment of the two processes, the GHG utility emissions in the case of the EO-MEG hydration process are three times more than the GHG utility emissions generated in the EC-MEG hydration process. In addition, the EC-MEG process is an environmentally friendly process that consumes carbon dioxide as a feedstock to react with ethylene carbonate to produce MEG.

In conclusion, the hydration process through EC as an intermediate is more feasible than the direct hydration process and it is a promising technology for ethylene oxide hydration that has been commercialized in several plants. This is due to the less energy consumption in the water separation section and the utilities GHG emissions compared to the EO-MEG process. However, more future research works are needed for both liquid phase oxidation and hydration through EC processes to enhance their economic benefits and make these processes more feasible than the EO/MEG process.

## Data Availability

All data generated or analyzed during this study are included in this published article.
